# The History of the Study of Clinical Medicine in Great Britain

**Published:** 1906-11-24

**Authors:** 


### The History of the Study of Clinical Medicine in Great Britain.

Dr. Fitzpatrick's bequest to the Royal College of Physicians of London is bearing good fruit. Dr. Payne's lectures on the early history of medicine in England were of the utmost value to students of the historical side of physic, and Dr. Moore's lectures given last week promise to be of at least equal importance. As the lectures are limited in number, Dr. Norman Moore wisely restricted himself on the present occasion to a consideration of the study of clinical medicine from the accession of the Stuart dynasty, though there is plenty of material at an earlier date for a whole course of lectures. Dr. Moore devoted some part of his first lecture to a consideration of the work of Sir Theodore Mayerne, a Doctor of Physic of Oxford, who was appointed first physician to King James I. In an age when physicians were so learned that they rarely observed for themselves and were dependent for their facts upon the observations of the inferior ranks of the profession—the barber-surgeons, and later the apothecaries—Sir Theodore was a brilliant exception. His observations were so clear and precise that it is possible to discover the diseases of which his patients died and the signs and symptoms from which they suffered. Dr. Moore says that Mayerne considered no point in his patient's history unworthy of consideration, and that he weighed the whole in relation to treatment and to prognosis. He began by a minute series of observations of the symptoms; then mentioned in succession the remedies which had been tried; discussed and determined the diagnosis and several points of the prognosis, and concluded by an elaborate statement of the treatment to be adopted. Mayerne attended Henry, Prince of Wales, during his fatal illness in 1612, and drew up such an excellent account of the symptoms, treatment, and post-mortem appearances, that it is fair to say, that nothing but the pathology of the period prevented him from being the first physician to recognise typhoid fever, of which there can be no reasonable doubt that the young prince died.

Mayerne's notes on Queen Henrietta Maria show equal care, and were written out in July 1641, when the queen was about to cross the sea "to cure her mind no less than her body." The note says that she had "some swelling of the liver and spleen, frequent swelling of the gums and painful teeth, several renal calculi, frequent cough, sleepless nights only soothed by syrup of poppy—never by laudanum—herpes of the upper lip, occasional inflammation of the right eye and of the eyelids, recurring headaches, curvature of the spine (scoliosis); the arm and hand of the right side thinner than those of the left, extreme general wasting and as regards affections of the mind (animi pathemata), anger violent but brief, long sadness, frequent tears." The details are carefully recorded, and, besides showing the excellence of Mayerne's clinical observation, present to us a picture of the Queen of Charles I., which, placed beside the lady so thin and pale with some grace, but no cheerfulness, in the canvasses of Vandyke, enables us to understand how her troubles in the world must have affected her, and leads us to judge leniently any defects of her manner or disposition, and to attribute them not to a fiendish nature, as did her political opponents, but in great part, at least, to a physical condition which must have greatly detracted from her enjoyment of life. It is clear from these and other similar pictures that the historian who would rewrite history from a human standpoint must take into account the physical weaknesses of the main actors in the drama of life before he can assign adequate reasons for their actions.

But Mayerne was not the only representative of clinical medicine, nor even the chief of the noble band who made medicine famous in the seventeenth century. Glisson, whose name is inseparably associated with the liver, took an equal interest in the study of rickets. His pamphlet on this disease appeared in 1650, and though it does not allude by name or number to particular patients, yet it shows by the precision of his statements that it rests upon many carefully noted observations. Sydenham was certainly greater than either Mayerne or Glisson as a clinical physician. He prided himself on studying diseases without any preconceived hypothesis and on recording plain matter of fact. He carefully guarded himself against being biassed by the hypothetical explanations current in his day. He would not accept the traditional classification of fevers derived from old Greek and Arabian medicine. He refused to regard fever as a mere process of fermentation, like the Chemical School; or as resulting from the collision of discordant particles, like the Mechanical School. But he was obliged to take something for granted, and he took as his base the medical system of Hippocrates. Sydenham advanced as far as the physics, the chemistry, and the physiology of his time would allow him; but he had neither the education nor the means to enlarge their bounds, and he was in consequence hampered by his environment. He erred less venially because he scarcely considered morbid anatomy but endeavoured to determine the species and ascertain the course and the treatment of diseases by clinical observation only.

"How admirable," says Dr. Norman Moore, "is Sydenham's account of measles, and, when it is compared with the books of his time and before, how original, how clearly he describes the onset and the method of appearance of the rash, and how well contrasts the circumstances which attend it with those of small-pox! 'The symptoms of the measles do not abate by the eruption as in the small-pox, yet I never observed the vomiting afterwards, but the cough and fever increase with the difficulty of breathing, weakness of the eyes and the defluction on them, with continual drowsiness and want of appetite as before.' His obvious originality is one reason for the great repute of his writings, and this originality is due not merely to his having thought differently, but also to his having seen more than his predecessors. Though Sydenham's is a general account, it is as distinctly based upon many clinical observations as if the notes of the cases he had seen were appended. Of the score of cases which he particularises, most are mentioned in illustration of points of treatment; but those of Thomas Chute, nephew of Lady Dacres, a young man with small-pox, and of Malthus the apothecary, who had a chronic arthritis, are excellent illustrations of his daily observations."

